# Supplementary material for: The association between plasma chemokines and breast cancer risk and prognosis: A mendelian randomization study
Source: Front Genet. 2023 Jan 4;13:1004931. doi: 10.3389/fgene.2022.1004931 (PMC9845285; doi:10.3389/fgene.2022.1004931)
Supplement: Supplementary file 1 [file Table2.DOCX]

**STROBE-MR checklist of recommended items to address in reports of Mendelian randomization studies**^1^ ^2^

| **Item No.** | **Section** | **Checklist item** | **Relevant text from manuscript** |
| --- | --- | --- | --- |
| 1 | **TITLE and ABSTRACT** | Indicate Mendelian randomization (MR) as the study’s design in the title and/or the abstract if that is a main purpose of the study | The association between plasmachemokines and breast cancer by molecular subtypes: A Mendelianrandomization study. |
|  | **INTRODUCTION** |  |  |
| 2 | **Background** | Explain the scientific background and rationale for the reported study. What is the exposure? Is a potential causal relationship between exposure and outcome plausible? Justify why MR is a helpful method to address the study question | In the first and second paragraphs of the introduction, we discuss why plasma chemokines are a potential causal exposure for breast cancer risk and prognosis. In the third and 4th paragraphs we discuss why Mendelian randomization is a reasonable approach to investigate a potential relationship between them. |
| 3 | **Objectives** | State specific objectives clearly, including pre-specified causal hypotheses (if any). State that MR is a method that, under specific assumptions, intends to estimate causal effects | In the last paragraphs of the introduction we describe the objectives of our study. |
|  | **METHODS** |  |  |
| 4 | **Study design and data sources** | Present key elements of the study design early in the article. Consider including a table listing sources of data for all phases of the study. For each data source contributing to the analysis, describe the following: | In the present study, we implemented a two-sample MR using summary statistics from genome-wide association studies (GWAS), we further used the UK Biobank and TCGA cohorts to reaffirm our findings.Data information were presented in the AVAILABILITY OF DATA AND MATERIALS section. |
|  | a) | Setting: Describe the study design and the underlying population, if possible. Describe the setting, locations, and relevant dates, including periods of recruitment, exposure, follow-up, and data collection, when available. | Available information about the GWAS studies is provided in the section “Methord Section”. Further information is given in each of the original GWAS publications. |
|  | b) | Participants: Give the eligibility criteria, and the sources and methods of selection of participants. Report the sample size, and whether any power or sample size calculations were carried out prior to the main analysis | The methods of selection of participants and the sample size calculations were carried out prior to the main analysis are described in "Study Populations" section of the Method section. |
|  | c) | Describe measurement, quality control and selection of genetic variants | Selection of genetic variants is described in the Methods section and more details are in sTable 1. |
|  | d) | For each exposure, outcome, and other relevant variables, describe methods of assessment and diagnostic criteria for diseases | We described the definition of each exposure factor in the selection of genetic instrumental variables for the methodology and provides the references on which the definitions are based. |
|  | e) | Provide details of ethics committee approval and participant informed consent, if relevant | All studies involved in the GWAS on the human plasma proteome and the Breast Cancer Association Consortium were approved by the relevant research ethics committee, and all participants provided informed consent. |
| 5 | **Assumptions** | Explicitly state the three core IV assumptions for the main analysis (relevance, independence and exclusion restriction) as well assumptions for any additional or sensitivity analysis | Described in the "Statistical Analysis" section of the methods. |
| 6 | **Statistical methods: main analysis** | Describe statistical methods and statistics used | Described in the "Statistical Analysis" section of the methods. |
|  | a) | Describe how quantitative variables were handled in the analyses (i.e., scale, units, model) | Described in the "Statistical Analysis" section of the methods. |
|  | b) | Describe how genetic variants were handled in the analyses and, if applicable, how their weights were selected | Described in the "Statistical Analysis" section of the methods. |
|  | c) | Describe the MR estimator (e.g. two-stage least squares, Wald ratio) and related statistics. Detail the included covariates and, in case of two-sample MR, whether the same covariate set was used for adjustment in the two samples | Described in the "Statistical Analysis" section of the methods. |
|  | d) | Explain how missing data were addressed | Described in the "Statistical Analysis" section of the methods. |
|  | e) | If applicable, indicate how multiple testing was addressed | Described in the "Statistical Analysis" section of the methods. |
| 7 | **Assessment of assumptions** | Describe any methods or prior knowledge used to assess the assumptions or justify their validity | We used R2 and F statistics to estimate the proportion of variance explained by SNPs. |
| 8 | **Sensitivity analyses and additional analyses** | Describe any sensitivity analyses or additional analyses performed (e.g. comparison of effect estimates from different approaches, independent replication, bias analytic techniques, validation of instruments, simulations) | A more detailed description of the sensitivity analysis is given in the "Statistical Analysis" section of the Method section. |
| 9 | **Software and pre-registration** |  |  |
|  | a) | Name statistical software and package(s), including version and settings used | All statistical tests were two-sided and performed using R 3.6.3 and Stata 15.1. |
|  | b) | State whether the study protocol and details were pre-registered (as well as when and where) | The analysis plan is described in the Method section. |
|  | **RESULTS** |  |  |
| 10 | **Descriptive data** |  |  |
|  | a) | Report the numbers of individuals at each stage of included studies and reasons for exclusion. Consider use of a flow diagram | Information is given in the "Study Population" section of the methods. |
|  | b) | Report summary statistics for phenotypic exposure(s), outcome(s), and other relevant variables (e.g. means, SDs, proportions) | Information is given in the "Study Population" section of the Method section. |
|  | c) | If the data sources include meta-analyses of previous studies, provide the assessments of heterogeneity across these studies | We give this information in the section of the Statistical Analysis |
|  | d) | For two-sample MR:  i.  Provide justification of the similarity of the genetic variant-exposure associations between the exposure and outcome samples  ii.  Provide information on the number of individuals who overlap between the exposure and outcome studies | All the sample populations are from Europe, so they are ethnically heterogeneous is very small, and the factors such as BMI and sex have been adjusted. |
| 11 | **Main results** |  |  |
|  | a) | Report the associations between genetic variant and exposure, and between genetic variant and outcome, preferably on an interpretable scale | In the main analysis, the selected SNPs explained 0.9%–36.3% of the variance in plasma chemokines, and the corresponding F-statistic ranged from 29.7–1879.3 (sTable 1), suggesting no evidence of weak instrument bias. |
|  | b) | Report MR estimates of the relationship between exposure and outcome, and the measures of uncertainty from the MR analysis, on an interpretable scale, such as odds ratio or relative risk per SD difference | Plasma CCL5 was causally associated with breast cancer in the MR analysis, which was significant in the luminal and HER-2 enriched subtypes and further confirmed using PRS analysis (OR=0.94, 95% CI=0.89-1.00). A potential causal association with breast cancer survival was only found for plasma CCL19, especially for ER-positive patients. In addition, we also found an inverse association between CCL19 expression in tumors and breast cancer overall and relapse-free survival (HR=0.58, 95% CI=0.35-0.95) |
|  | c) | If relevant, consider translating estimates of relative risk into absolute risk for a meaningful time period | We give this information in the Results section. |
|  | d) | Consider plots to visualize results (e.g. forest plot, scatterplot of associations between genetic variants and outcome versus between genetic variants and exposure) | Figure 1-2 are visualized results. |
| 12 | **Assessment of assumptions** |  |  |
|  | a) | Report the assessment of the validity of the assumptions | Sensitivity analysis using MR Egger and weighted median methods yielded similar results (sTable 3). |
|  | b) | Report any additional statistics (e.g., assessments of heterogeneity across genetic variants, such as *I^2^*, Q statistic or E-value) | sTable 2 |
| 13 | **Sensitivity analyses and additional analyses** |  |  |
|  | a) | Report any sensitivity analyses to assess the robustness of the main results to violations of the assumptions | Sensitivity analysis using MR Egger and weighted median methods yielded similar results (sTable 3 and sTable4). |
|  | b) | Report results from other sensitivity analyses or additional analyses | Sensitivity analysis using MR Egger and weighted median methods yielded similar results (sTable 3 and sTable4). |
|  | c) |  | For those identified plasma chemokines in the two sample Mendelian randomization studies for breast cancer, a single-sample Mendelian randomization was further applied to validate the findings in UK Biobank. |
|  | d) | When relevant, report and compare with estimates from non-MR analyses | We did validation study using gene expression data from TCGA. |
|  | e) | Consider additional plots to visualize results (e.g., leave-one-out analyses) | Fig 1 and Fig 2 |
|  | **DISCUSSION** |  |  |
| 14 | **Key results** | Summarize key results with reference to study objectives | We describe key results in the first paragraph of the discussion section. |
| 15 | **Limitations** | Discuss limitations of the study, taking into account the validity of the IV assumptions, other sources of potential bias, and imprecision. Discuss both direction and magnitude of any potential bias and any efforts to address them | Discussion paragraph 6 |
| 16 | **Interpretation** |  |  |
|  | a) | Meaning: Give a cautious overall interpretation of results in the context of their limitations and in comparison with other studies | Our analysis found an inverse association between CCL5 and breast cancer, with a similar effect size in a recent phenome-wide Mendelian randomization. |
|  | b) | Mechanism: Discuss underlying biological mechanisms that could drive a potential causal relationship between the investigated exposure and the outcome, and whether the gene-environment equivalence assumption is reasonable. Use causal language carefully, clarifying that IV estimates may provide causal effects only under certain assumptions | Discussion paragraph 2,3,4. |
|  | c) | Clinical relevance: Discuss whether the results have clinical or public policy relevance, and to what extent they inform effect sizes of possible interventions | Discussion paragraph 3,4,5,6. |
| 17 | **Generalizability** | Discuss the generalizability of the study results (a) to other populations, (b) across other exposure periods/timings, and (c) across other levels of exposure | In the discussion section, we discuss potential caveats in terms of generalizing our findings. |
|  | **OTHER INFORMATION** |  |  |
| 18 | **Funding** | Describe sources of funding and the role of funders in the present study and, if applicable, sources of funding for the databases and original study or studies on which the present study is based | We have reported all sources of funding in the manuscript. |
| 19 | **Data and data sharing** | Provide the data used to perform all analyses or report where and how the data can be accessed, and reference these sources in the article. Provide the statistical code needed to reproduce the results in the article, or report whether the code is publicly accessible and if so, where | We give access information to all data used in the study in the Generated Statement: Publicly available datasets were analyzed in this study. And we also provide the version of Rand Stata. |
| 20 | **Conflicts of Interest** | All authors should declare all potential conflicts of interest | All authors have declared conflicts of interest (none reported). |

This checklist is copyrighted by the Equator Network under the Creative Commons Attribution 3.0 Unported (CC BY 3.0) license.

1. Skrivankova VW, Richmond RC, Woolf BAR, Yarmolinsky J, Davies NM, Swanson SA, et al. Strengthening the Reporting of Observational Studies in Epidemiology using Mendelian Randomization (STROBE-MR) Statement. JAMA. 2021;under review.

2. Skrivankova VW, Richmond RC, Woolf BAR, Davies NM, Swanson SA, VanderWeele TJ, et al. Strengthening the Reporting of Observational Studies in Epidemiology using Mendelian Randomisation (STROBE-MR): Explanation and Elaboration. BMJ. 2021;375:n2233.
